# Network analysis of surgical innovation: Measuring value and the virality of diffusion in robotic surgery

**DOI:** 10.1371/journal.pone.0183332

**Published:** 2017-08-25

**Authors:** George Garas, Isabella Cingolani, Pietro Panzarasa, Ara Darzi, Thanos Athanasiou

**Affiliations:** 1 Surgical Innovation Center, Department of Surgery and Cancer, Imperial College London, St. Mary’s Hospital, London, United Kingdom; 2 Department of Surgical Research and Innovation, The Royal College of Surgeons of England, London, United Kingdom; 3 Big Data and Analytical Unit, Imperial College London, St. Mary’s Hospital, London, United Kingdom; 4 School of Business and Management, Queen Mary University of London, London, United Kingdom; Dalian University of Technology, CHINA

## Abstract

**Background:**

Existing surgical innovation frameworks suffer from a unifying limitation, their qualitative nature. A rigorous approach to measuring surgical innovation is needed that extends beyond detecting simply publication, citation, and patent counts and instead uncovers an implementation-based value from the structure of the entire adoption cascades produced over time by diffusion processes. Based on the principles of evidence-based medicine and existing surgical regulatory frameworks, the surgical innovation funnel is described. This illustrates the different stages through which innovation in surgery typically progresses. The aim is to propose a novel and quantitative network-based framework that will permit modeling and visualizing innovation diffusion cascades in surgery and measuring virality and value of innovations.

**Materials and methods:**

Network analysis of constructed citation networks of all articles concerned with robotic surgery (n = 13,240, Scopus^®^) was performed (1974–2014). The virality of each cascade was measured as was innovation value (measured by the innovation index) derived from the evidence-based stage occupied by the corresponding seed article in the surgical innovation funnel. The network-based surgical innovation metrics were also validated against real world big data (National Inpatient Sample–NIS^®^).

**Results:**

Rankings of surgical innovation across specialties by cascade size and structural virality (structural depth and width) were found to correlate closely with the ranking by innovation value (Spearman’s rank correlation coefficient = 0.758 (p = 0.01), 0.782 (p = 0.008), 0.624 (p = 0.05), respectively) which in turn matches the ranking based on real world big data from the NIS^®^ (Spearman’s coefficient = 0.673;p = 0.033).

**Conclusion:**

Network analysis offers unique new opportunities for understanding, modeling and measuring surgical innovation, and ultimately for assessing and comparing generative value between different specialties. The novel surgical innovation metrics developed may prove valuable especially in guiding policy makers, funding bodies, surgeons, and healthcare providers in the current climate of competing national priorities for investment.

## Introduction

Innovation has long occupied center stage in the medical and health sciences. In surgery, it has been held up as a catalyst of unprecedented advances that have led to substantial improvements in healthcare delivery and patient outcomes [1]. A variety of surgery-specific innovation frameworks have been proposed, among which the IDEAL is the most widely implemented paradigm that categorizes surgical innovation into distinct stages [2]. Most existing frameworks, however, suffer from a unifying limitation. Their qualitative nature has thwarted comparative assessments of the innovation-generating potential of individual academic surgeons-scientists, academic surgical research groups, institutions, countries, and surgical specialties. Moreover, earlier attempts to assess surgical innovation solely through publication, citation, and patent counts were premised on the oversimplifying assumption of equating innovation value with short-lived surges in popularity, and failed to capture the long-term impact of innovation upon healthcare delivery [3].

To address these shortcomings, we propose a novel and quantitative network-based framework for measuring the value of surgical innovation [4]. To this end, we leverage on the structure of the adoption-induced fingerprints produced by diffusion processes as they unfold over time [5]. Indeed, innovation typically triggers complex diffusion processes, driven by social contagion mechanisms, in which individuals’ adoption is a function of their exposure to others’ knowledge, attitude, or behavior. Diffusion can therefore be mapped out as a time-varying cascade of adoptions that propagate from individual to individual over potentially many generations of adopters. Any attempt to capture how increasingly popular innovations ultimately transform medical care ought to explicitly account for the size, structural depth, and breadth of the whole adoption cascade underpinning diffusion [6].

This article aims to make a contribution in this direction by using citation networks to study the structural foundations of innovation diffusion in surgery. Drawing on a unique and comprehensive dataset on robotic surgery, we propose a novel set of network-based measures for uncovering the virality of adoption cascades. We demonstrate how these network measures facilitate comparative assessments of different robotic surgical procedures in terms of how they diffuse and implement innovation. Our framework can therefore play a fundamental role in guiding and assisting policy-makers, funding bodies, and healthcare providers.

## Materials and methods

### Dataset

Drawing upon SciVerse Scopus^®^ (Elsevier^®^, Amsterdam, The Netherlands) [7], we extracted all articles concerned with robotics (i.e., containing the MeSH terms ‘robot’ and ‘robotic’ in their title, abstract, or keywords) across all surgical specialties from the start of the database (1974) until December 2014. Moreover, we limited our dataset to articles published in scientific journals, and restricted the scope of the analysis to the subject area ‘Medicine’. Therefore, any article that did not represent original research studies on robotic surgery was excluded (e.g., review article or conference article).

**Fig 1:**
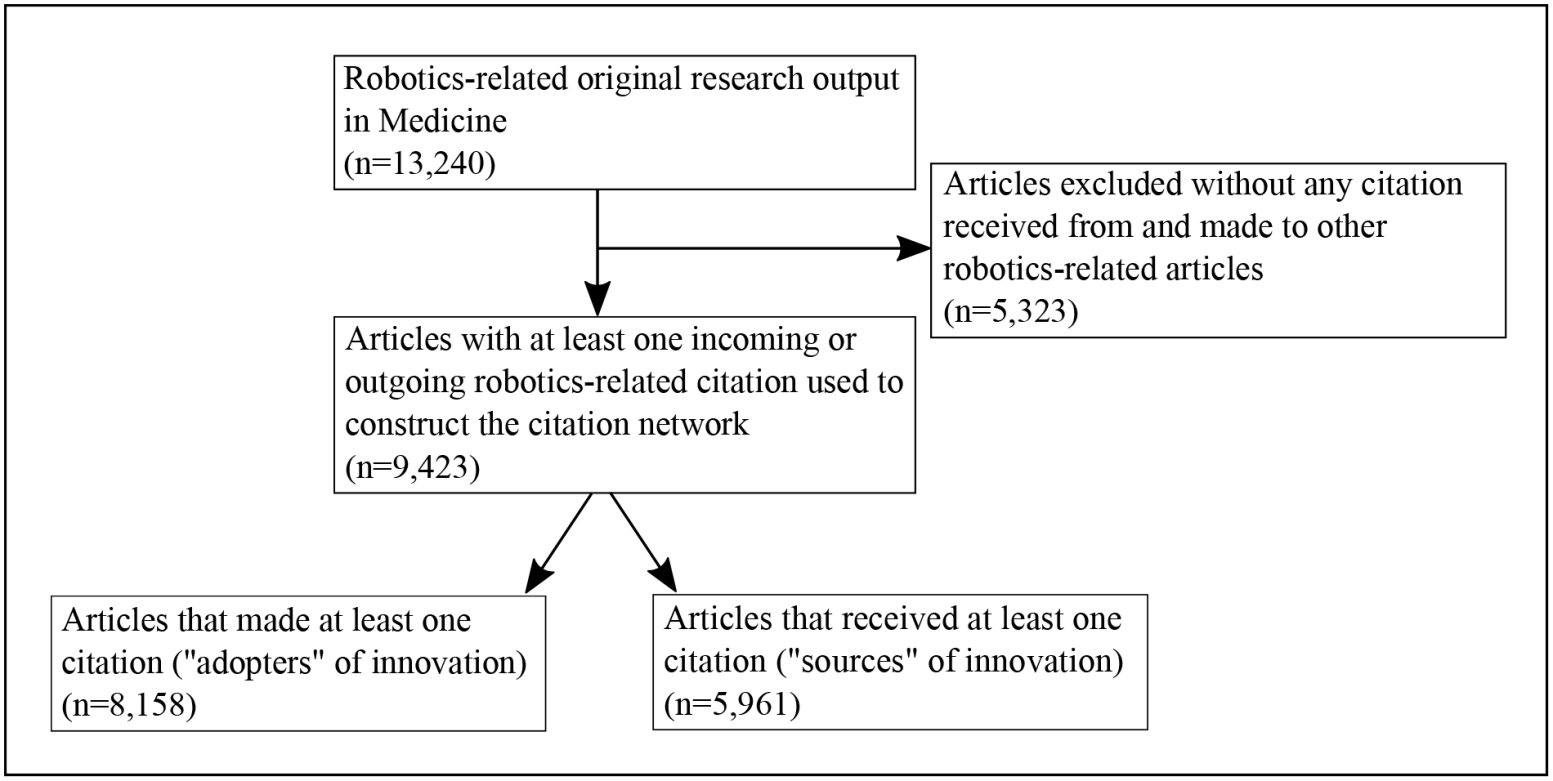
Flow diagram illustrating the search strategy used for generating the citation networks. Note that the intersection between the two sets of articles at the bottom used to construct the citation networks is non-empty as an article can both cite and be cited.

The initial search produced 13,240 publications, of which 9,423 were articles that either received or made at least one citation within the broad medical field of robotics. Among these, 5,961 articles received at least one citation from another article within the field, and can therefore be regarded as ‘sources’ of innovation, whereas 8,158 articles made at least one citation to another article within the field, and can therefore be regarded as ‘adopters’ of innovation (Fig 1).

**Fig 2:**
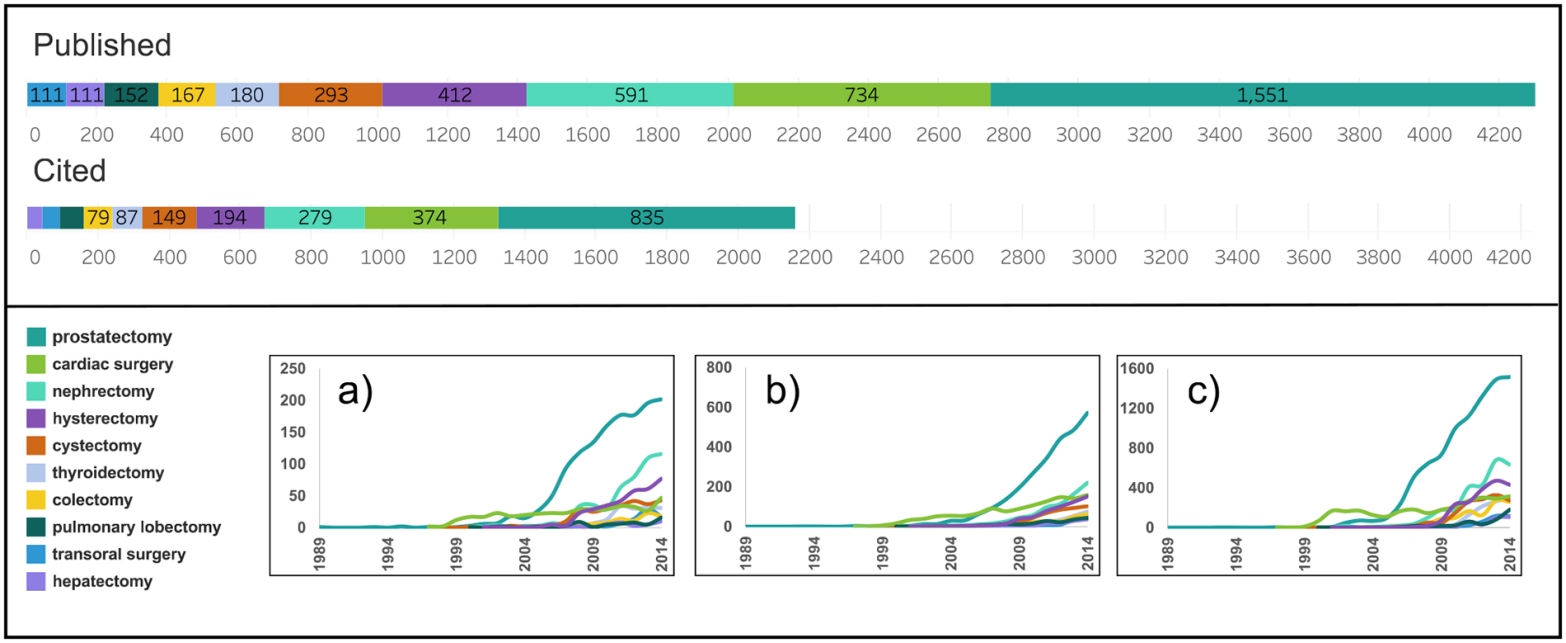
Scientific production and citations across surgical specialties. Published and cited articles per surgical specialty (top panel). Cumulative number of published and cited articles: (a) number of published articles over time, (b) number of cited articles over time, (c) number of citations received over time by published articles.

Using the MeSH terms reported in S1 Online Supplement, we allocated each of the 5,961 articles that received at least one citation to one of 16 categories (based on surgical specialty and/or procedure). We limited our study to the ten specialties (the terms specialties and procedures are used interchangeably) with at least 100 of the original 13,240 publications. In total, the final dataset includes 4,918 articles. Among these, there are 2,159 articles across the ten specialties that received at least one citation, and 2,759 articles that do not necessarily belong to any of the ten specialties, but are part of chains of citations leading to articles in those specialties (Fig 2 and S1 Online Supplement).

### Citation networks and diffusion cascades

In a citation network, the nodes are the articles, and a directed link is established from one article to another if the former cites the latter in its bibliography [8, 9]. A citation network can therefore be thought of as a diffusion cascade along which information spreads and adoption of innovation propagates.

**Fig 3:**
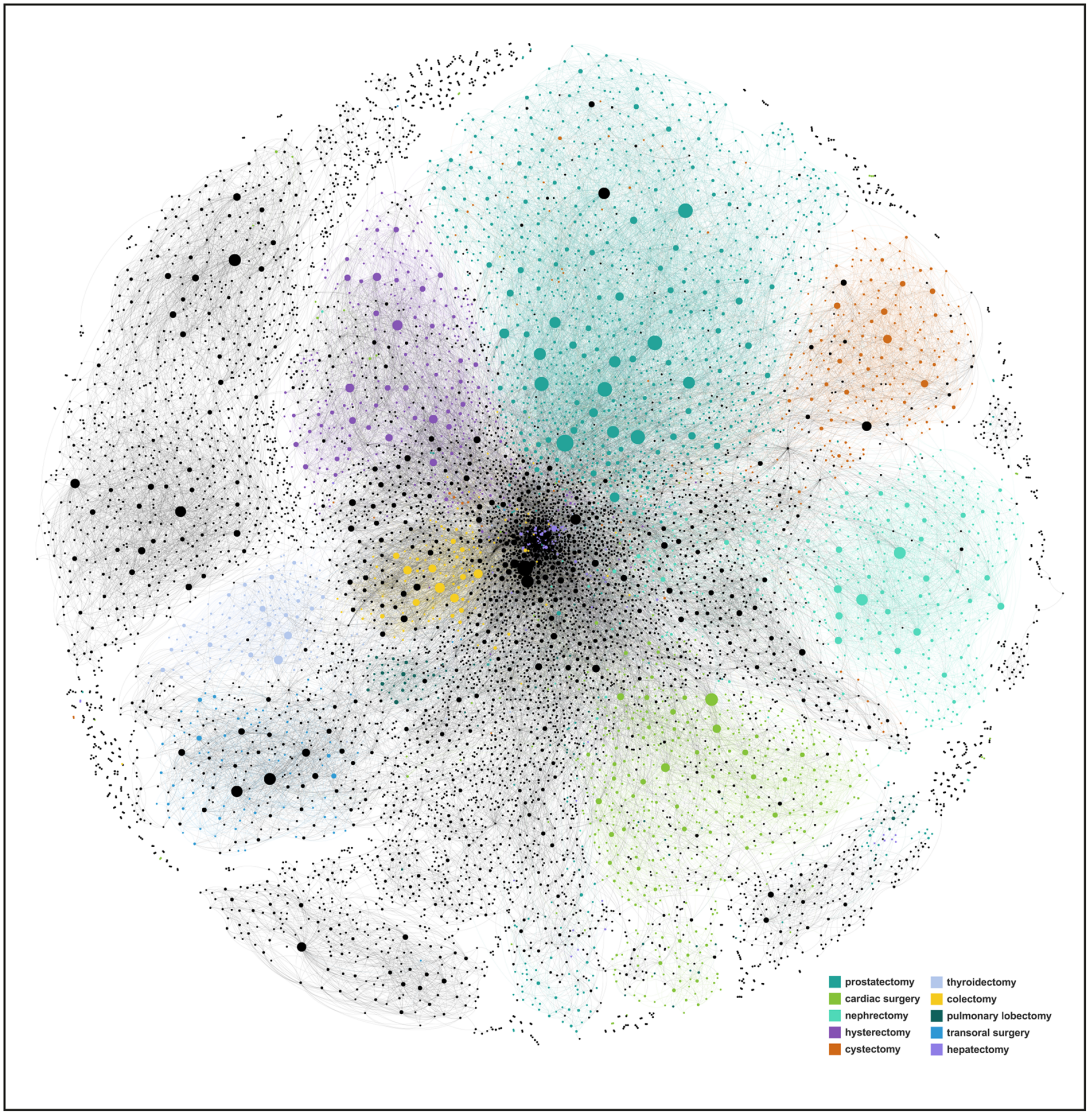
Network of citations. The network includes 9,423 articles that received or made at least one citation. The visualization of the network was obtained through the network visualization software Gephi^®^ (Gephi^®^ Consortium, Compiegne, France). The size of each node *i* is proportional to its in-degree $k_{i}^{in}$ (i.e., the number of citations received), and the color associated with each node denotes the surgical specialty it belongs to. The network is partitioned into topological communities that are coextensive with surgical specialties (S1 Online Supplement).

For each of the 9,423 articles that received or made at least one citation, we constructed the diffusion cascade based on the corresponding citation network (Fig 3). In these cascades, each citation-based chain of adoption can be traced back to the seed node representing the original article in which a given innovation was introduced (as an idea description or laboratory evaluation) in the first place. The structure of these cascades can therefore shed light on the depth and breadth of the diffusion process through which innovations, once proposed, built up momentum over time [10].

### Measuring broadcast and viral diffusion processes: Citations, cascade size, structural depth, and structural width

**Fig 4:**
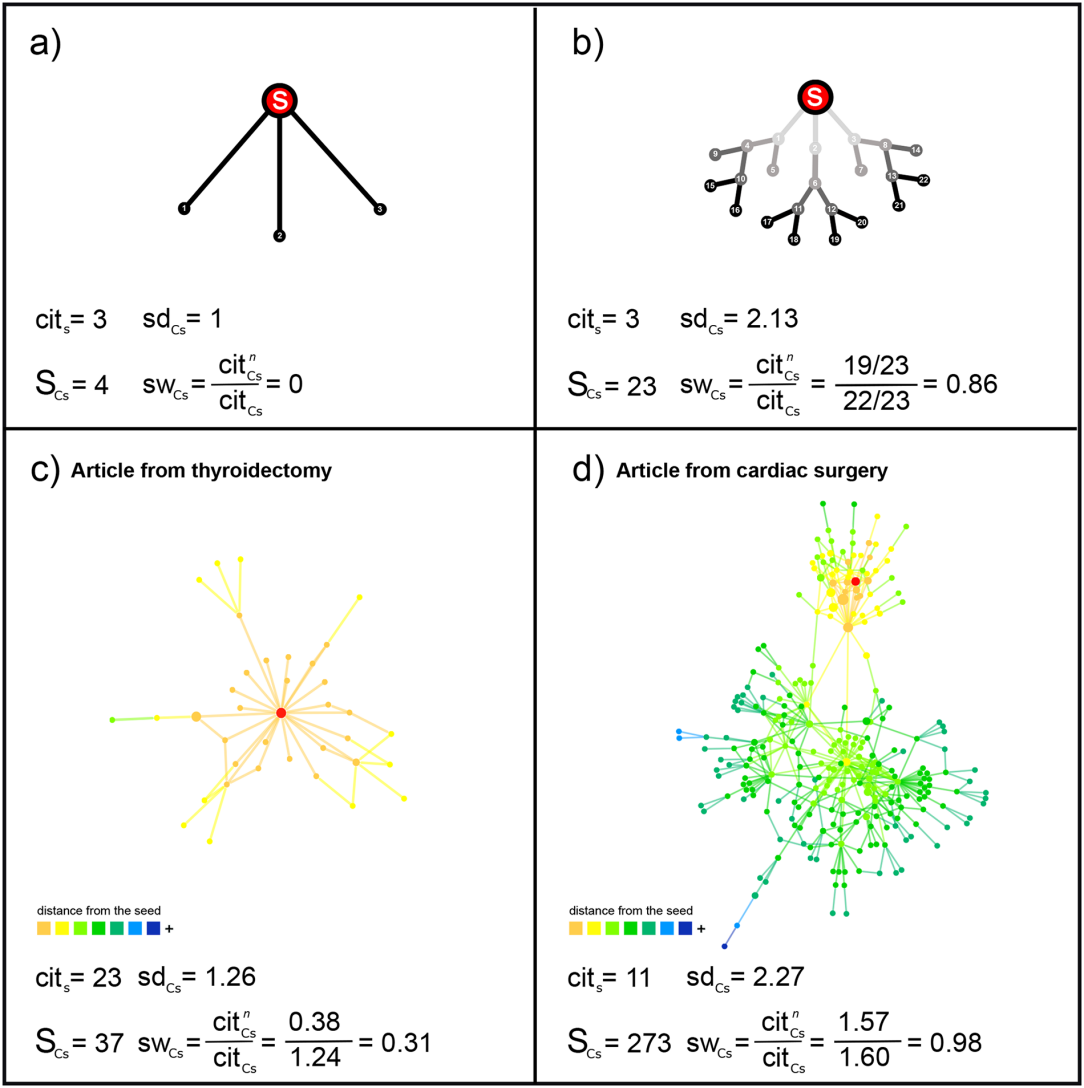
Schematic representation of cascades and real world examples. Top panels: (a) broadcast-driven diffusion; (b) viral diffusion. In both cases, we calculated the number of citations received by the seed node, the size of the cascade, the structural depth, and the structural width of the cascade. Bottom panels: two real examples of cascades within (c) thyroidectomy and (d) cardiac surgery. For each cascade, we calculated the number of citations to seed article, the cascade size, and structural depth and width. The color of nodes denotes their distances from the seed article (red node). Links between nodes carry the color of the citing node. The size of each node is proportional to the ratio between the node’s second-step citations and first-step citations.

Popularity can be gained through two main modes of diffusion: broadcast and viral spreading (Fig 4, panels a and b). While broadcast spreading is dominated by processes of bursty adoptions from a single seed article, viral spreading is typically characterised by multi-generational branching processes in which any article receives a citation from only few others, thus yielding multiple, widespread, and long chains of citations extending over time [5].

To investigate broadcast-driven diffusion processes, we quantified two measures. First, for each seed article *s* that received at least one citation, we calculated the total number of citations *Cit*_*s*_ received from any other article in the corresponding cascade *C*_*s*_. Second, for each seed article *s*, we calculated the fraction of citations *Cit*_*s*,*year*_ received from any other article in *C*_*s*_ within one year since the date of publication of *s*.

To uncover the virality of diffusion processes, we computed three measures (S1 Online Supplement). First, for each seed article *s*, we calculated the size $S_{C_{s}}$ of the corresponding cascade *C*_*S*_, namely the total number of articles in *C*_*S*_ (including seed article *s*). Second, drawing on a classical graph property and recent work on online diffusion [11], we propose a measure for quantifying the multi-generational nature of adoption cascades. Specifically, for each seed article *s* we define the structural depth $sd_{C_{s}}$ of cascade *C*_*S*_, as the average length of the shortest directed paths between pairs of articles in *C*_*s*_. Formally, for *n>1* articles,

$$sd_{C_{s}}=\frac{1}{l}\sum_{i\in C_{s}}\sum_{j\in C_{s}}d_{\underset{i,j}{\to }},\;i\neq j,$$

where $d_{\underset{i,j}{\to }}$ denotes the length of the directed shortest path from article *i* to article *j*, *C*_*s*_ is the set of nodes belonging to the cascade originating from article *s*, and *l* is the number of directed paths connecting pairs of articles in *C*_*s*_. So constructed, $sd_{C_{s}}$ becomes larger as the adopters of the original idea proposed by seed article *s* are farther apart from one another and from *s*, thus producing a multi-generational cascade. An important property of structural depth is that it varies as a function of the size of the cascade only under certain conditions (S1 Online Supplement).

Finally, we introduce a measure for quantifying the branching structure of a diffusion cascade [5, 6, 12, 13]. Specifically, we define the structural width $sw_{C_{s}}$ of cascade *C*_*s*_ as the ratio between the average number of second-step citations ($cit_{C_{s}}^{n}$) and the average number of citations ($cit_{C_{s}}$) accrued by articles in *C*_*s*_. Formally, for n>*1* articles,

$$sw_{C_{s}}=\frac{cit_{C_{s}}^{n}}{cit_{C_{s}}}.$$

Thus, the larger $sw_{C_{s}}$, the more widespread and locally dense the diffusion cascade is. In particular, $sw_{C_{s}}$ ranges between zero in the limiting case of the star graph (with no second neighbors) and indefinitely large values in cases of branching cascades with highly widespread tree-like local structures.

Combined, cascade size, depth and width enable us to capture the multi-faceted nature of viral innovation [5, 6, 12, 13]. While cascade size captures the overall popularity of innovation based on total number of adopters, structural depth and width shed light on how popularity is gained. Specifically, depth captures the multi-generational character of diffusion processes. In this sense, innovation becomes viral not simply because it is widely adopted (typically, as a result of extensive media coverage and large advertising efforts), but because it propagates further beyond the first generation of initial adopters. Finally, structural width uncovers the branching tree-like character of diffusion. In this sense, innovation becomes viral when a large population of adopters ‘infect’ a much larger population, with ripple effects producing a rapid, large-scale increase in popularity as in viral disease spreading. Thus, these three measures jointly quantify virality as a function of: (a) the overall number of adopters; (b) the number of generations of adopters; and (c) the contribution of each adopter to overall diffusion. This enables us to create ranking lists from the most viral innovations to those that only generate short-term surges in popularity and then quickly die out. Fig 4 (panels c and d) shows two real cascades that differ in structural virality.

### Measuring the value of surgical innovation: The implementation-based innovation index

**Fig 5:**
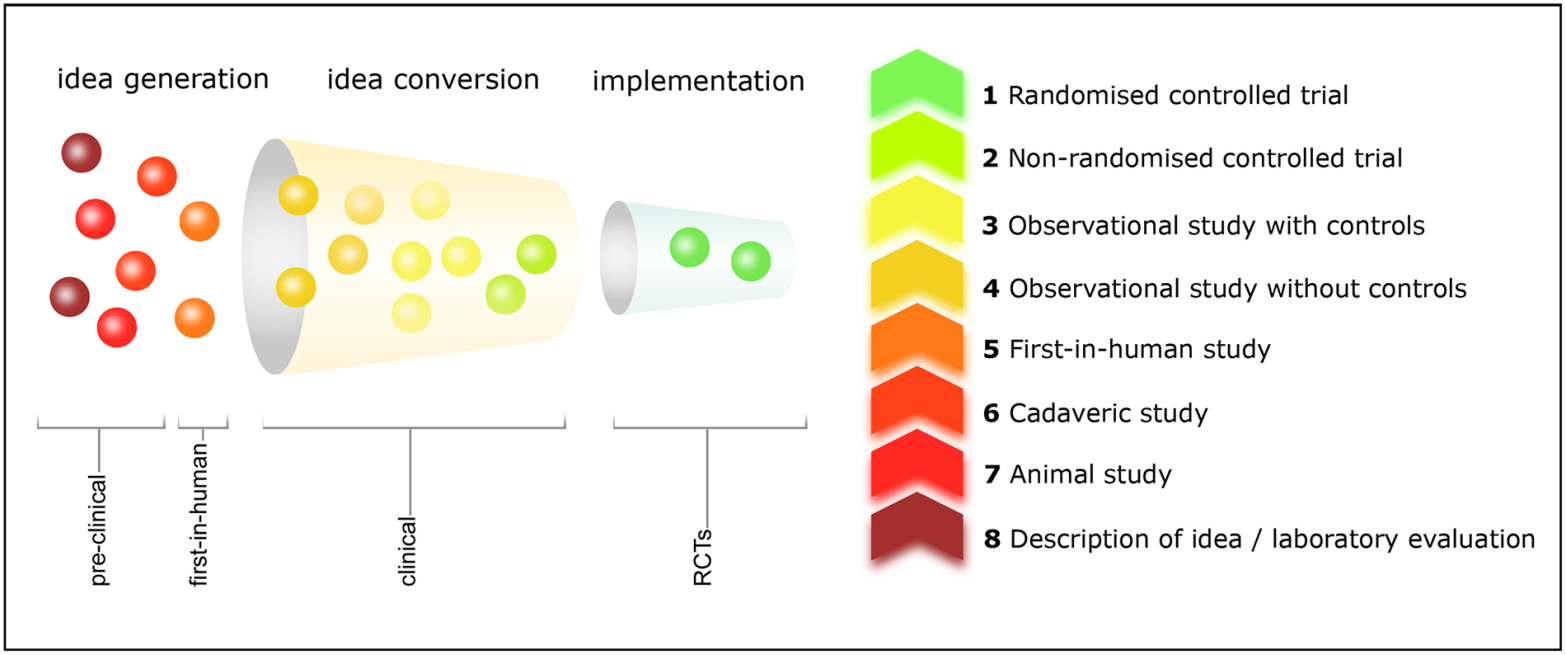
The surgical innovation funnel. Left-hand panel: the surgical innovation funnel illustrating the different stages through which innovation in surgery typically progresses. Right-hand panel: the different stages of innovation implementation according to the level of evidence.

We propose a novel metric aimed at capturing the intrinsic value of a surgical innovation as a function of the degree to which it has reached an implementation stage. To this end, a score was attributed to each seed article based on its corresponding level of evidence (S1 Online Supplement) [14]. The US Department of Health and Human Services evidence levels were used as they only include numerical values (with no lettered subcategories) facilitating classification [15]. Further stages of implementation were added for pre-clinical categories (description of idea/laboratory evaluation, animal, and cadaveric studies), as illustrated in the surgical innovation funnel (SIF) in Fig 5. The SIF shows that innovation follows a trajectory akin to natural selection whereby as “the going gets tough, the tough get going” (i.e., only the fittest among attention-seeking ideas will survive as they move along the SIF) [16, 17]. All studies were scored by two independent academic surgeons-scientists (G.G. and T.A.), and disagreements arbitrated by a third academic surgeon-scientist (A.D.) Formally, for each surgical specialty *g∈G*, we define the innovation index as

$$i_{g}=\frac{\sum_{c\in S}\frac{1}{c}\times \frac{p_{c}^{g}}{P_{c}}}{\underset{g\in G}{\text{max}}\left(\sum_{c\in S}\frac{1}{c}\times \frac{p_{c}^{g}}{P_{c}}\right)}\;\times \;100,$$

where *c* ∈ [1, …, 8] is an integer value labeling the ordinal category associated with the implementation stage according to level of evidence, $p_{c}^{g}$ is the count of publications by surgical specialty *g* appearing in category *c*, and $P_{c}=\sum_{g}p_{c}^{g}$ is the total number of publications appearing in category *c* across all surgical specialties in *G* (S1 Online Supplement).

To validate our innovation index, additional data from the National (Nationwide) Inpatient Sample (NIS^®^) database were collected and ranked in terms of the actual numbers of robotic procedures performed in the US in 2012 (most recent publicly available database when this study was conducted). As NIS^®^ approximates a 20% stratified sample of all discharges from US community hospitals containing big data from more than seven million hospital stays per year, it can reasonably be used to provide empirical support in favour of findings based on our innovation index (S1 Online Supplement) [18].

## Results

### Ranking surgical innovations by broadcast-driven popularity

**Fig 6:**
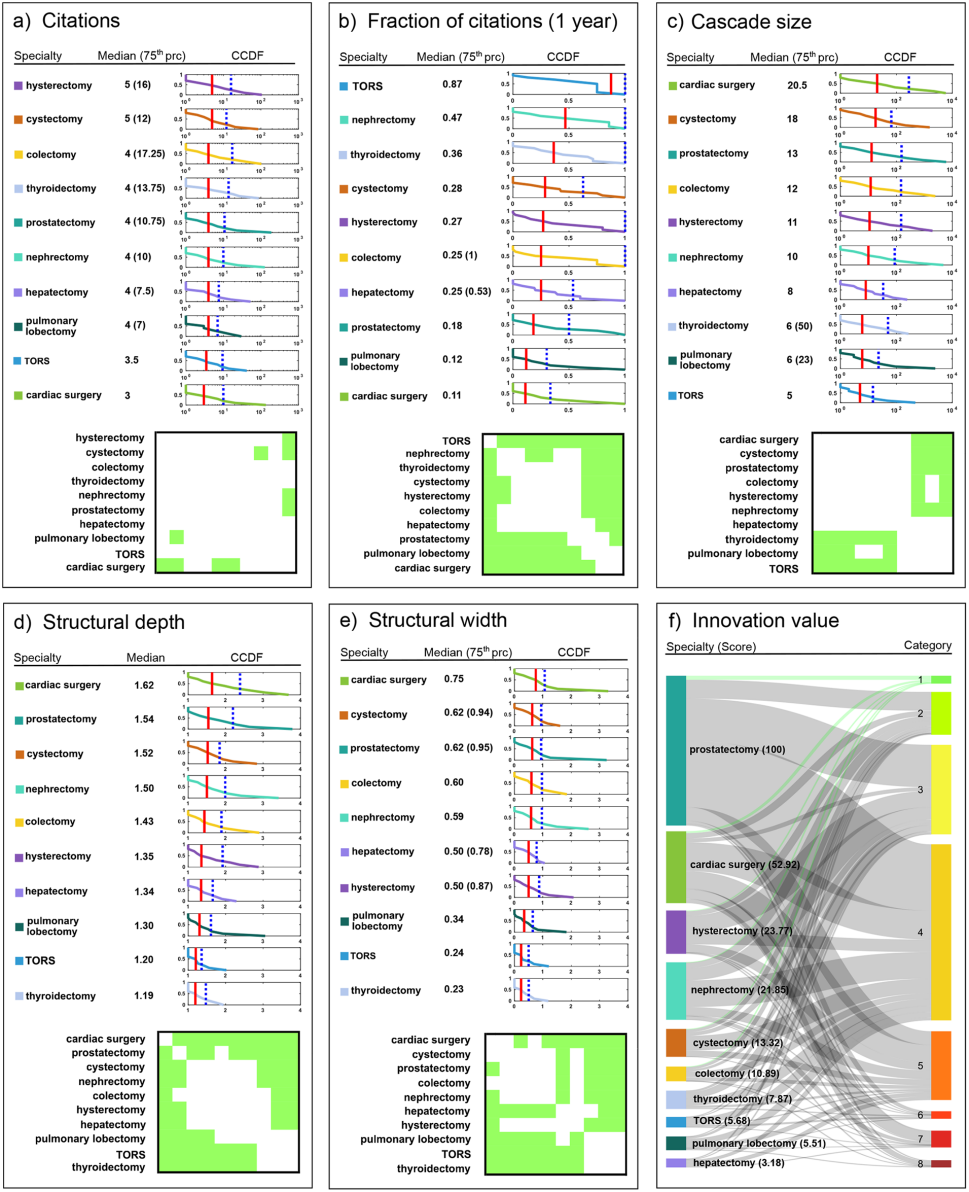
Rankings of surgical specialties. (a) Ranking by citations to seed articles; (b) ranking by fraction of citations to seed articles within one year; (c) ranking by cascade size; (d) ranking by structural depth; (e) ranking by structural width. Rankings were obtained by using the medians (red lines) of the complementary cumulative distribution functions (CCDFs). In case of ties between medians, the 75^th^ percentiles (dotted blue lines) were used. The bottom of each panel shows results from Mann-Whitney U tests (for citations and size) and Kolmogorov-Smirnov tests (for fraction of citations, depth, and width) of independence between pairs of distributions (green color: p<0.05). For clarity, specialties are reported only as labels to the rows of the square matrix, but are equally listed as labels to the columns from left to right. (f) Sankey diagram representing the ranking of surgical specialties in terms of innovation index. Specialties are listed in the left column, with the top-ranked scoring the highest by innovation index. The height of colored bars is proportional to the number of published articles in the corresponding specialty. Levels of evidence are listed in the right column, with the top-ranked being the final implementation stage. The height of colored bars is proportional to the number of published articles at the corresponding level of evidence across all specialties. The width of the lines connecting a given specialty to a given level of evidence is proportional to the number of articles published by the specialty at that level of evidence. Green lines refer to contributions of specialties to the final stage of implementation.

Fig 6 shows the rankings of robotic surgical procedures in terms of the medians of the distributions of: (i) citations received by seed articles (panel a); and (ii) fractions of citations received by seed articles within one year since publication (panel b). Results from Mann-Whitney U (for citations) and Kolmogorov-Smirnov (for fraction of citations) tests for comparing pairs of such distributions are not statistically significantly different.

### Ranking surgical innovations by virality

For each robotic surgical procedure, we measured the size, structural depth and width of each cascade, and produced the corresponding frequency distributions. We then ranked these in terms of the medians of such distributions (Fig 6, panels c, d, e). Cardiac surgery occupies the top of the rankings, followed by urological procedures, specifically prostatectomy, cystectomy, and nephrectomy. Mann-Whitney U test (for size) and Kolmogorov-Smirnov tests (for depth and width) were used for comparing each pair of distributions (S1 Online Supplement). Cascade size, structural depth, and width of cardiac surgery are statistically significantly different from those of all other specialties, as occurs with second-ranking prostatectomy (except when compared to colectomy). At the other end of the ranking list, thyroidectomy occupies the last position, overtaken by transoral robotic surgery (TORS) though the difference between the two does not reach statistical significance (p>0.05). Notice that differences between any of the three highest-ranking specialties and any of the three lowest-ranking ones are all statistically significant (p<0.05).

### Ranking surgical innovations by evidence-based innovation value

Fig 6 (panel f) reports a Sankey diagram illustrating the contribution of each surgical specialty/procedure to each level of evidence, and the ranking by innovation index (S1 Online Supplement). Results suggest that prostatectomy ranks first with the greatest number of Randomized Controlled Trials (RCTs), followed by cardiac surgery, hysterectomy, nephrectomy and cystectomy. At the bottom of the ranking list are thyroidectomy, TORS, pulmonary lobectomy, and hepatectomy. To validate our measure of innovation, we compared the ranking of innovation across surgical specialties by innovation index with the ranking based on real world evidence from the NIS^®^ database, and found a statistically significant correlation [19, 20]. To account for the small sample size, we calculated exact p-values and obtained: Spearman’s rank correlation coefficient = 0.673, p = 0.039; Kendall’s tau coefficient = 0.511, p = 0.047.

### Comparisons across rankings of surgical specialties

The ranking of robotic surgical procedures by innovation index closely matches the rankings by cascade size, structural depth, and width. We tested the similarity between these three pairs of rankings by evaluating the Kendall’s concordance coefficient based on exact p-values to account for the small sample size. We obtained: 0.879 (p = 0.007), 0.891 (p = 0.005), 0.812 (p = 0.030), respectively for the tests of independence between rankings by innovation index and cascade size, innovation index and structural depth, innovation index and structural width (S1 Online Supplement). Interestingly, no statistically significant correlation (p>0.05) was found between ranking by either citations or fraction of citations received within one year, on the one hand, and ranking by either cascade size, structural depth or width, on the other (S1 Online Supplement). Most importantly, there is no statistically significant correlation (p>0.05) between the ranking by either citations or fraction of citations within one year, on the one hand, and the ranking by innovation index on the other (S1 Online Supplement). We also tested the similarity between all pairs of distributions by calculating exact p-values and adjusting for multiple comparisons based on false discovery rate (FDR) correction, and obtained similar results (S1 Online Supplement).

## Discussion

Despite the prominent role of innovation in surgery, only limited attention has been paid to quantifying its value as a function of diffusion processes and implementation. An effective way to model the global spread of ideas, knowledge, and innovation is through network analysis [21, 22]. Network analysis has been previously used in surgical research but only in the context of network meta-analysis and scientific collaboration networks [23–25]. This is the first study to employ network analysis for the measurement of surgical innovation in terms of value and virality of diffusion.

Our study has made a three-fold contribution in this direction. First, we proposed a novel measure for the value of surgical innovation that directly accounts for the evidence-based implementation stage reached in clinical practice. The ranking of surgical specialties by this measure was found to closely match the one based on big data derived from the real word (i.e., the NIS^®^ database). For example, it is no coincidence that robotic prostatectomy and cardiac surgery, widely supported by RCTs, have the highest score in the ranking, while robotic thyroidectomy, characterized by a remarkably poor uptake in the Western world, occupies the lowest position [26]. Second, we introduced a novel network-based framework for assessing the structure of adoption cascades. Specifically, we proposed measures for quantifying the virality of these cascades. Third, we demonstrated that the ranking of robotic surgical procedures by innovation value positively correlates with rankings by virality, but not with rankings by broadcast-driven popularity.

We focused on robotic surgery for a number of reasons. First, it is a sufficiently recent innovative technology so as to play a salient role in most surgeons’ work, at least in the Western world. Second, it also boasts a sufficiently long history so as to enable the tracing of adoption cascades over time. Moreover, as the robotic surgery market is dominated by the da Vinci^®^ surgical robot (Intuitive Surgical^®^, Inc., Sunnyvale, CA), it lies at the interface between different surgical specialties, most of which use different versions of the same robot (e.g., standard, S, Si, Xi), thus providing a ‘common comparator’ when it comes to ranking (the same) innovation as applied to different surgical specialties [27].

Our results can have a number of implications for research, clinical practice, and policy. First, by using citation networks to uncover the footprints of adoption behavior, our study opens up new avenues for future work on knowledge transfer and sharing, and the way healthcare providers discover, combine, and apply new information over time. Second, our study complements and extends existing frameworks for surgical innovation, and enables suitable combinations of quantitative and qualitative assessments of innovation value. Third, our framework can be easily extended to also produce rankings of institutions, research centers, academic surgical groups, and even countries (i.e., at the macro level), and equally, at the other end of the spectrum (i.e., at the micro level), produce rankings of individual academic surgeons-scientists in terms of their ability and potential to produce pioneering innovation. From this perspective, our network-based framework can play a fundamental role in guiding policy [28, 29], strategically directing medical research funding, and assisting healthcare providers in their efforts to optimize resource allocation and improve the quality of healthcare delivery and patient outcomes [30]. For example, our findings suggest that policy-makers should focus on whether recently introduced innovations (e.g., 3D printing, percutaneous valve implantation technology, and augmented reality for intraoperative navigation in robotic surgery), in spite of their limited adoption history, have managed to produce highly viral diffusion cascades, and thus have the potential of redirecting clinical practice [31, 32].

Most importantly, our findings on short-term broadcast-driven popularity should alert healthcare providers and policy makers to the dangers of using mere citation counts as predictors of value-generating potential. Seemingly successful research efforts, with a disproportionally large number of citations, may quickly die out leaving no influential trace over time. By contrast, research that is only mildly successful in the short run may well gain in popularity over time, yield wide and multi-generational cascades of adoptions, and ultimately provide the foundation for successful medical practice.

Therefore, our study suggests that surgical innovations that have started producing viral adoption cascades should be further sustained, as they are likely to prove highly successful in the end, even if they are still at their initial stage of development and diffusion. As such, the structural virality metric can act as a forecasting tool for surgical innovation.

On the other hand, surgical innovations that only produce broadcast-like adoption cascades or cascades with low virality values should not be further sustained in an attempt to unlock some (hidden) potential of value generation as these represent surgical innovations that in fact are not likely to prove successful or of practical value.

A number of limitations in our study should be noted. First, citation networks represent but one type of innovation networks. Other networks include co-authorship and collaboration networks where nodes represent individual surgeons-scientists or institutions. Drawing on these networks, one could investigate the structural foundations of innovation at the individual level, and the relationship between scientific collaboration and innovation diffusion. Second, we did not assess whether the citation made to an innovation was positive or negative as a citation can be made to level criticism to the content of an article. This, however, is beyond the scope of the current study, and will certainly be addressed in our future work. Third, our measures do not directly account for variations in time scales across cascades. Innovations may trigger cascades of adoption stretching over various intervals of time, but nonetheless characterized by the same degree of virality. Future work shall extend our measures to also account for temporal variations in adoption behavior. Finally, a broader family of network metrics, including degree correlations among adjacent nodes, will be required to fully assess the structure of adoption cascades and the multi-faceted value of surgical innovation.

In conclusion, our study has demonstrated that network analysis offers unique new opportunities for understanding, modeling and measuring surgical innovation, and ultimately for assessing and comparing the generative value in the different specialties. When evidence-based data are difficult to collect or not yet available, a suitable methodological substitute is needed to inspire and guide the decisions of policy makers, funding bodies, surgeons, and healthcare providers. This is especially relevant in the current financial climate characterized by paucity of funding in both health-related research and healthcare delivery combined with competing national priorities for investment. Our study constitutes an important first step in this direction.

## Supporting information

S1 Online Supplement (DOCX)
