# Supplementary material for: Network analysis of surgical innovation: Measuring value and the virality of diffusion in robotic surgery
Source: PLoS One. 2017 Aug 25;12(8):e0183332. doi: 10.1371/journal.pone.0183332 (PMC5571947; doi:10.1371/journal.pone.0183332)
Supplement: S1 Online Supplement — (DOCX) [file pone.0183332.s001.docx]

# **S1 Online Supplement. Supporting information.**

# **S1. Data**

# **S1.1 The construction of the citation networks**

To construct the network of citations among articles concerning robotic surgery, we searched in Elsevier’s Scopus^®^ database for all those articles related to robotic surgery. In total, we retrieved 14,775 articles that were assigned to the major subject area “Medicine”, including “robotic” in the title or in the abstract or in the keywords, and were published from 1974 until the 19th of December 2015.

In Scopus^®^, articles can be assigned to one or more major subject areas according to the classification of the journal where they have been published. Journals are typically assigned to one or more subject areas of different granularity. In total, there are 27 major subject areas, and 313 specific subject categories according to the Scopus^®^ Subject Areas and Subject Categories. The scientific journals that belong to the major subject area “Medicine” represent 34% of all journals included in Scopus^®^ (last update in May 2017). Notice that all journals included in the MEDLINE database are also included in Scopus^®^. Keywords, when available, included those selected directly by the authors of articles and those assigned manually by a Scopus^®^ team of professional indexers, according to the MeSH vocabulary and the EMTREE medical terms. Typically, Scopus^®^ adds index terms to about 80% of articles included in the Scopus^®^ database.

Since the Scopus^®^ database only included part of the articles published in 2015, we decided to filter out from our data set the 1,504 articles published in that year. Moreover, our original data set included 31 articles expected to be published in 2016 that we decided not to include. Once it was cleaned and filtered as described above, the data set included a total of 13,240 articles.

Given these 13,240 articles of interest, we collected the unique electronic identificators (EIDs) used by Scopus^®^ database to uniquely identify them. We then used the EIDs to retrieve the information needed to build an acyclic directed network of citations. For each of the 13,240 articles that received at least one citation, we retrieved the EIDs of the articles that made a citation to the focal article during the period starting from the year of the publication of the article up to the 31^st^ of December 2014. In particular, we considered only citations originating from articles pertaining to robotics in Medicine and published by the end of 2014 (i.e., belonging to our set of 13,240 articles). In this way, we restricted our focus to the citation network among articles only within robotics in Medicine. Indeed, for example, if any given article among the set of 13,240 ones had received citations only from articles not belonging to this set of 13,240 articles or from articles pertaining to robotics in Medicine but published after the 31^st^ of December 2014, then this article would be associated with a zero count of citations. On the one hand, among the 13,240 articles, 5,961 received at least one citation from an article belonging to the same set (i.e., 5,961 articles have an in-degree equal to or higher than one). On the other, 8,158 articles among the 13,240 ones have made at least one citation to another article belonging to the same set (i.e., 8,158 article have an out-degree equal to or higher than one). A total of 9,423 articles out of the 13,240 ones are connected to the others through at least one incoming or outgoing citation.

In total, our data set includes 4,918 articles. Among these, there are 2,159 articles across ten surgical specialties (see section S2.2) that received at least one citation. Each of these 2,159 articles represents the seed of a diffusion cascade. In addition, the data set includes 1,049 articles across the ten specialties that did not receive any citations, but are part of chains of citations leading to articles in those specialties. Finally, the data set includes 1,710 articles that do not belong to any of the ten specialties, but are part of chains of citations leading to articles in those specialties.

**S1.2 Visualisation of the citation network**

Fig 3 in the main text shows the network of citations among the 9,423 articles that are connected to other articles through at least one incoming or outgoing citation. For unweighted networks, it is common practice to attribute unitary weight to the links. The algorithm used to visualize the networks is called ForceAtlas2^®^, which is the default layout algorithm [1]. It implements an attraction-repulsion model aimed at simulating a physical system: unconnected nodes pull each other further apart, while links between connected nodes push the nodes closer to each other. The process of physically locating nodes depends only on connections between nodes, and the final result varies according to the initial state. Possible exogenous or endogenous attributes of nodes are not taken into account in locating them. The position of each node cannot be interpreted on its own, but needs to be compared with the position of the other nodes, depending on the whole structure of connection. The energy model that underpins the ForceAtlas2^®^ algorithm is based on two simple rules: (a) the attraction between two nodes depends linearly on the distance between them; and (b) the repulsion force between any two nodes is inversely proportional to the distance between them, and is directly proportional to the product of their degrees. In this way, repulsion is weaker between a very connected node and a poorly connected node than between two very connected nodes. Given that in our specific application we are considering binary networks with unitary weights, the distance between any two connected nodes is set to be equal to one, and is constant across all connections in each network. Unlike other algorithms, and as a result of the interplay between the attraction and repulsion forces implemented, ForceAtlas2^®^ produces densely populated spatial regions that identify structural communities. Moreover, ForceAtlas2^®^ comes with a set of optional settings that can be used by users to optimize the visualization of networks. One of the settings used to depict networks is the “scaling” constant that is applied to stretch homogenously all the dyadic repulsion forces between nodes in order to expand the network distances without affecting the relative positioning of the nodes in the layout. We set the “scaling” constant at a value equal to two (default is one). In this way, the image becomes more readable, thus enabling an interpretation of the networks. We also use the “Gravity” option, and set the parameter at 70 (default is one). This prevents disconnected components from being pulled apart from each other. Moreover, it enables nodes within these components to be placed toward the center of the visualized network. Finally, we used the “LinLog Mode” option that applies a logarithmic function to the attraction force. This creates a visualization of the network that maximizes the modularity of the community structure.

**S2. Methods**

# **S2.1 Partition of articles into groups related to surgical specialties**

We clustered the articles into 17 groups. Among these, 16 correspond to 16 surgical specialties/procedures: prostatectomy, cardiac surgery, nephrectomy, hysterectomy, cystectomy, thyroidectomy, colectomy, pulmonary lobectomy, TORS, hepatectomy, esophagectomy, gastrectomy, cholecystectomy, skull base surgery, transplantation and microvascular surgery. In addition, we created a group of articles that did not fall into any of the above 16 groups. In order to partition the articles into these groups, we searched for those articles containing words in their titles that uniquely identify the various surgical operations. For example, for robotic prostatectomy we identified 1,551 articles within the broad field of robotic surgery that contained “prostatectomy” or “prostate” in their titles.

We then selected ten of the 16 surgical specialties/procedures corresponding to those with a number of publications higher than 100 out of the original 13,240 articles that belong to the major subject area “Medicine” and relate to robotics. In particular, the following six specialties were filtered out: cholecystectomy, esophagectomy, gastrectomy, transplantation, skull base surgery, and microvascular surgery. In total, our study focused on the following ten surgical specialties/procedures: prostatectomy, cardiac surgery, nephrectomy, hysterectomy, cystectomy, thyroidectomy, colectomy, pulmonary lobectomy, TransOral Robotic Surgery (TORS), and hepatectomy. Fig 2 in the main text shows that these ten groups of articles related to the robotic-assisted surgical specialties evolve over time in different ways.

We applied a pattern matching technique (Regex^®^) for allocating each article among the 5,961 that received at least one citation to one of the main 16 surgical specialties, i.e., cardiac surgery, cholecystectomy, colectomy, cystectomy, esophagectomy, gastrectomy, hepatectomy, hysterectomy, microvascular surgery, nephrectomy, prostatectomy, pulmonary lobectomy, skull base surgery, TORS, transplantation, and thyroidectomy.

Specifically, we identified all those articles containing in their title word(s) or combination(s) of words related to the above mentioned 16 surgical specialties/procedures as detailed in S1 Table. Regex^®^ (or regular expression) is a language for logical pattern matching. It is chiefly used to produce text patterns matching a given text. For example, using the regular expression language when we searched for all the titles containing either “prostatectomy” or “prostate” followed by or preceded by any text, we searched for a pattern written as “prostat\w*”. The expression “\w***” after “prostat” (the common text pattern between the two words “prostatectomy” and “prostate”) is meant to represent all the possible combinations of characters following the common string pattern. The symbol “|” in a regular expression stands for the logical conjunction “or”, meaning that, for example, in “cystectomy| bladder” we searched for all titles containing either the word “cystectomy” or the word “bladder”.

Moreover, as occurred for example in the case of cardiac surgery, we have identified a set of words uniquely associated with all those articles pertaining to this specialty. Among them, there is also a combination of two words such as “coronary” and “bypass” that can be also combined with “artery” to form “coronary artery bypass”. In order to search for both “coronary bypass*”* and “coronary artery bypass” we used the string pattern “coronary.*bypass” that matches all those patterns within the title starting with the word “coronary*”*, ending with the word “bypass*”*, and (although not necessarily) containing some other object (e.g., word, space, hyphen) between those two.

Finally, to address possible differences in language, as for example in the case of esophagectomy, depending on whether the authors use British English or American English, we can distinguish between titles containing “oesopha\w***” or “esopha\w***” as a common string pattern followed by any string of characters. In this case, the regular expression to use is “o?esopha\w***”.

**S1 Table.** Regular expressions used to cluster articles into groups related to surgical specialties.

| **Surgical specialty/procedure** | **Regular expressions** |
| --- | --- |
| **Cholecystectomy** | " cholecystectomy\| gallbladder" |
| **Cardiac surgery** | " heart\| coronary\| coronary.*bypass\| cardiac\| valve\| atrial septal defect\| atrial fibrilation\| ablation\| pacemaker leads\| mitral\| aortic" |
| **Colectomy** | " colectomy\| colorectal\| colonic\| ca?ec\w*\| hemicolectomy\| right\| transverse\| left\| sigmoid\w*\| rectum\| anterior resection" |
| **Cystectomy** | " cystectomy\| bladder" |
| **Esophagectomy** | " o?esopha\w* |
| **Gastrectomy** | " gastrectomy\| stomach\| sleeve" |
| **Hepatectomy** | " hepatectomy\| liver\| hepatic" |
| **Hysterectomy** | " hysterectomy\| salpingo-oopherectomy\| uterus\| fibroid\| endometri\w* " |
| **Microvascular surgery** | " free.*flap\| microvascular" |
| **Nephrectomy** | " nephrectomy\| renal" |
| **Prostatectomy** | " prostat\w*" |
| **Pulmonary lobectomy** | " pulmonary.*lobectomy\| lung.* lobectomy\| bronch\w*.*lobectomy\| lung\| pulmonary\| bronch\w*" |
| **Skull base surgery** | " skull.*base\| pituitary" |
| **Thyroidectomy** | " thyroid\w*" |
| **TransOral Robotic Surgery (TORS)** | " transoral.*robot\| TORS" |
| **Transplantation** | " transplant\w*" |

# **S2.2 Analytical definition of measures**

**S2.2.1 Citations**

In the citation network, the nodes are the articles, while a directed link originates from one article to another when the former cites the latter. As citations can only point backward in time, from more recently published articles to older ones, there are no closed loops of directed links in citation networks. As such, these networks are termed ‘acyclic’ [2].

Each article in the network is characterized by the count of citations received from other articles belonging to the network that includes all published articles pertaining to robotic surgery. So constructed, the number of citations received by an article corresponds to the in-degree of the corresponding node in the citation network, i.e., the number of links pointing to that node. Let us define $\boldsymbol{A}=[a_{ij}]$ as the adjacency matrix describing the connections among all the articles (nodes) in the network, such that the entry $a_{ij}$ is equal to one if there is a directed link (citation) from *i* to *j*, and zero otherwise. Thus, the count of citations *cit_i_* received by an article *i* or, equivalently its in-degree $k_{i}^{in}$in the network of citations, can be defined as follows

$${cit}_{i}= k_{i}^{in}= \sum_{j} a_{ji} .$$

**S2.2.2 Cascades, structural depth, and structural width**

Any cited article can be considered a seed node *s* from which a cascade *C_s_* originates. We define a cascade *C_s_* as the set of nodes including the seed node *s* and all nodes connected to node *s* through direct or indirect links.

To calculate structural depth, for each seed node *s*, we computed all shortest directed paths originating from each pair of nodes belonging to the cascade *C_s_*. Given two nodes *i* and *j* belonging to *C_s_*, the shortest directed path from node *i* to node *j* is given by the length of the shortest sequence of directed links that separate node *i* from node *j*^2^. We denote with $d_{\vec{i,j}}$ the length of the directed shortest path from node *i* to node *j* within a given cascade, and with *l* the number of all directed shortest paths connecting all pairs of nodes in the cascade. Note that, given the directed nature of our network, the existence of a shortest directed path from node *i* to node *j* does not necessarily imply that a shortest path between node *j* and node *i* also exists. Therefore, because in a citation network paths are always directed backwards in time, and thus closed loops are not allowed, each pair of articles *i* and *j* contributes only once to the value of ${sd}_{C_{s}}$to the extent that a directed path exists from article *i* to article *j*, or vice versa.

Our measure of structural depth ${sd}_{C_{s}}$draws on, and extend, a classical graph property, the Wiener index, that was originally proposed in mathematical chemistry [3]. The application of the Wiener index to online diffusion cascades was recently introduced by Goel et al. [4]. Our definition of structural depth builds upon this analytical tradition. Like the measure of structural virality proposed by Goel et al. [4], our measure takes into account all shortest paths; however, unlike the Wiener index and Goel et al.’s structural virality, our measure of structural depth explicitly accounts for the direction of links. In particular, given the acyclic directed nature of the network of citations, we chose to focus only on directed shortest paths between pairs of articles.

An important property of structural depth is that it varies as a function of the size of the cascade only under certain conditions. Specifically, in the case of a stylized tree-like cascade in which each article is cited by at most *k* other articles (i.e., a *k-*ary rooted tree), ${sd}_{C_{s}}$will increase with the total number of articles *N* in the cascade only when *k << N*. Indeed, when *k << N* is kept unchanged, an increase in *N* will amplify the number of generations in the cascade, thus increasing ${sd}_{C_{s}}.$ In particular, in a complete *k*-ary tree-like cascade (i.e., a cascade in which each article that is not a leaf is cited by *k* other articles, and all articles that are leaves have the same length), ${sd}_{C_{s}}$will be proportional to the height of the cascade. Conversely, when *k ≈ N*, an increase in *N* will only produce larger broadcasts, with virtually no effect upon ${sd}_{C_{s}}$(approaching the value of one in the limiting case of the star graph).

To calculate the structural width ${sw}_{C_{s}}$of cascade *C_s_*, for each article *i* in *C_s_* (including seed article *s*), we computed ${cit}_{i}^{n}$, namely the number of citations received by all articles citing article *i*, and then calculated the average of such values over all articles in *C_s_*. To obtain ${cit}_{C_{s}}$, for each article *i* in *C_s_* (including seed article *s*), we calculated *cit_i_*, namely the number of citations received by *i*, and then calculated the average of such values over all articles in *C_s_*.

Fig 4 (panels a and b) in the main text shows two simple cases that differ in terms of their cascade structure. Even though the two seed nodes have the same number of citations, they are associated with different values of structural depth and width. S2 Table illustrates how the lengths of the shortest paths were calculated on each of the two cases. S3 Table illustrates how citations and second-step citations were calculated for each node in each of the cascades. S4 Table shows how citations, structural depth and structural width were computed in each of the two examples.

**S2 Table.** Count of shortest paths by length in the cases in Fig 4 (panels a,b; main text).

| **Case (a)** | | **Case (b)** | |
| --- | --- | --- | --- |
| **Length of shortest path** | **Count** | **Length of shortest path** | **Count** |
| 1 | 3 | 1 | 22 |
| 2 | 0 | 2 | 19 |
| 3 | 0 | 3 | 14 |
| 4 | 0 | 4 | 8 |

**S3 Table.** Count of citations and second-step citations in the examples in Fig 4 (panels a,b; main text).

| **Case (a)** | | | **Case (b)** | | |
| --- | --- | --- | --- | --- | --- |
| **Node** | **Citations** | **Second-step citations** | **Node** | **Citations** | **Second-step citations** |
| s | 3 | 0 | s | 3 | 5 |
| 1 | 0 | 0 | 1 | 2 | 2 |
| 2 | 0 | 0 | 2 | 1 | 2 |
| 3 | 0 | 0 | 3 | 2 | 2 |
|  |  |  | 4 | 2 | 2 |
|  |  |  | 5 | 0 | 0 |
|  |  |  | 6 | 2 | 4 |
|  |  |  | 7 | 0 | 0 |
|  |  |  | 8 | 2 | 2 |
|  |  |  | 9 | 0 | 0 |
|  |  |  | 10 | 2 | 0 |
|  |  |  | 11 | 2 | 0 |
|  |  |  | 12 | 2 | 0 |
|  |  |  | 13 | 2 | 0 |
|  |  |  | 14 | 0 | 0 |
|  |  |  | 15 | 0 | 0 |
|  |  |  | 16 | 0 | 0 |
|  |  |  | 17 | 0 | 0 |
|  |  |  | 18 | 0 | 0 |
|  |  |  | 19 | 0 | 0 |
|  |  |  | 20 | 0 | 0 |
|  |  |  | 21 | 0 | 0 |
|  |  |  | 22 | 0 | 0 |

**S4 Table.** Citations, structural depth, and structural width in two examples in Fig 4 (panels a,b; main text).

| **Measure** | **Case (a)** | **Case (b)** |
| --- | --- | --- |
| **Citations** | 3 | 3 |
| **Structural depth** | 3/3=1 | (1×22+2×19+3×14+4×8)/63=2·127 |
| **Structural width** | [(0×4)/4]/[(0×3+3×1)/4] = (0/4)/(3/4) = 0 | [(0×16+2×5+4×1+5×1)/23]/[(0×12+1×1+2×9+3×1)/23] = (19/23)/(22/23)=0·86 |

Below we report values of structural depth for each of the examples in Fig 4 (panels a,b; main text) calculated using the formula introduced by Goel *et al*. [4], and compare them with the ones calculated with our measure (structural depth).

**S5 Table.** Structural virality based on Goel *et al*. [4] and structural depth.

| **Measure** | **Case (a)** | **Case (b)** |
| --- | --- | --- |
| **Structural virality** | (2×6+1×6)/12 = 1·5 | (1×44+2×62+3×48+4×56+5×80+6×88+7×80+8×48)/506=4·76 |
| **Structural depth** | 3/3=1 | (1×22+2×19+3×14+4×8)/63=2·13 |

**S2.3 Measuring innovation**

**S2.3.1 The innovation funnel and the innovation value chain**

Innovation can hardly be defined unequivocally across domains. In particular, in the context of surgery, innovation may relate to the manufacturing and advancement of medical devices, improvement in surgical technique or novel approach, change in healthcare service delivery process, marketing intervention, or organizational reconfiguration that must be “altogether new, new to anatomic location, and/or new to the category of patient” [5].

Our study has benefitted from the use of two concepts originally applied to industrial innovation: the innovation funnel and the innovation value chain [6, 7]. The surgical innovation funnel (SIF) we describe depicts the process along which new ideas, once introduced (idea generation), progress until only those that are successful in being materialized can reach the stage of conversion or translation (e.g., manufacturing of a new medical device, performance of a first-in-human surgical technique or approach, or introduction of a new marketing intervention or healthcare service delivery model in surgical practice). As the funnel further narrows, many of these ‘materialized’ innovations will die out while a select minority will continue until they reach the final implementation stage (i.e., usage of a new medical device or surgical technique in a number of patients but confined within a trial and/or institution). If implementation succeeds and the innovation is widely employed, then the diffusion process can be regarded as having been reached (the final stage of implementation). For instance, this occurs with the FDA-approval and market release of a new medical device, the international uptake of a new surgical technique, or the introduction of a new healthcare service delivery model in surgical practice. This progressive ‘natural selection’ process underlying the innovation value chain has inspired our measure of innovation value, the innovation index, that is meant to express the influential role of a surgical innovation as a function of the degree to which it has reached the final stage of implementation.

**S2.3.2 Innovation index**

In order to assess the level of success reached by innovative ideas in the ten groups defined by type of surgical specialties/procedures, we partitioned innovation into eight distinct stages corresponding to eight ordered categories, numbered in descending order from eight to one (S6 Table). Each article can be classified according to the method and level of evidence it presents. In particular, the least mature stage in the generic innovation path is the theoretical description of an idea or its laboratory evaluation (stage eight). An idea can then be applied and assessed through an animal study (stage seven), and subsequently through a cadaveric study (stage six), and so on along all the stages described in Fig 5 in the main text. The initial idea, if successful, will end up being employed in a Randomized Controlled Trial (stage one), which represents the final implementation stage at which an innovation can be regarded as having reached the highest level of evidence in medical practice. For each surgical specialty, we quantified the average progress of innovative ideas towards their final implementation stage using what we have defined as the innovation index (main text).

**Table S6.** Description of surgical innovation stages into which articles can be classified according to their level of evidence.

| **Surgical Innovation Stage** | **Description** |
| --- | --- |
| **1** | Randomized controlled trial |
| **2** | Non-randomized controlled trial |
| **3** | Observational study with controls |
| **4** | Observational study without controls |
| **5** | First-in-human study |
| **6** | Cadaveric study |
| **7** | Animal study |
| **8** | Description of idea / laboratory evaluation |

The value of the innovation index based on the eight stages of innovation varies from zero to 2.718. In principle, the maximum value is reached by a surgical specialty that manages to monopolize the whole production of articles across all surgical innovation stages. In this limiting case, the values for any category *c* are equal to one, and the innovation index corresponds to a harmonic series with eight terms, converging to 2.718. We then normalized the value of the innovation index by dividing it by the maximum that was observed across all surgical specialties, i.e., 1.11. The normalized values will then range between zero and one. We then multiplied the normalized values by 100 to obtain percentage values.

To understand the logic behind the innovation index, let us consider the simple case in which we have only two surgical specialties. The first specialty (*g=*1) accounts for 2/3 of the whole scientific production at each stage from eight to five, and for 1/3 of the whole production at each stage from four to one. The second group (*g=*2) accounts for 1/3 of the production at each stage from eight to five, and for 2/3 of the production at each stage from four to one (see S7 Table for details). The innovation index $i_{1}$for group 1 is then equal to 70, while the innovation index $i_{2}$ for group 2 is equal to 100. This means that group 2 contributes more than group 1 to relatively mature stages of innovation in surgery. As a result, group 2 is more successful than group 1 in bringing innovations to the stage at which they have implications for medical practice (e.g. in terms of policy or clinical decision-making) and thus create value for patients. S8 Table illustrates how the contributions to each stage are combined into a final normalized value of the innovation index.

**S7 Table.** Calculation of contributions to implementation stages in two illustrative examples.

| **Category** | ***c*** | *8* | *7* | *6* | *5* | *4* | *3* | *2* | *1* |
| --- | --- | --- | --- | --- | --- | --- | --- | --- | --- |
| **Weight** | ***1/c*** | 1/8 | 1/7 | 1/6 | 1/5 | 1/4 | 1/3 | 1/2 | 1/1 |
| **Contribution Group 1** |  | 2/3 | 2/3 | 2/3 | 2/3 | 1/3 | 1/3 | 1/3 | 1/3 |
| **Contribution Group 2** |  | 1/3 | 1/3 | 1/3 | 1/3 | 2/3 | 2/3 | 2/3 | 2/3 |

**S8 Table.** Calculation of innovation index in two illustrative examples.

| **Group 1** | $\sum_{c\in S} \frac{1}{1}\times\frac{1}{3}+\frac{1}{2}\times\frac{1}{3}+\frac{1}{3}\times\frac{1}{3}+\frac{1}{4}\times\frac{1}{3}+\frac{1}{5}\times\frac{2}{3}+\frac{1}{6}\times\frac{2}{3}+\frac{1}{7}\times\frac{2}{3}+\frac{1}{8}\times\frac{2}{3}$ = 1.12 | (1.12/1.6) ×100 | 70 |
| --- | --- | --- | --- |
| **Group 2** | $\sum_{c\in S} \frac{1}{1}\times\frac{2}{3}+\frac{1}{2}\times\frac{2}{3}+\frac{1}{3}\times\frac{2}{3}+\frac{1}{4}\times\frac{2}{3}+\frac{1}{5}\times\frac{1}{3}+\frac{1}{6}\times\frac{1}{3}+\frac{1}{7}\times\frac{1}{3}+\frac{1}{8}\times\frac{1}{3}$ = 1.6 | (1.6/1.6) ×100 | 100 |

# **S2.3.3 National Inpatient Sample (NIS®) count of robotic procedures in 2012**

The 7,296,968 hospital discharges included in the National Inpatient Sample (NIS**®**) 2012 data set have been extracted according to the ICD-9-CM**®** procedure codes within the principal procedure field (PR1), as shown in S9 Table.

**S9 Table.** ICD-9-CM**®** codes for the selection of principal surgical procedures in NIS**®** 2012.

| **Surgical procedure** | **ICD-9-CM Codes for principal procedures** |
| --- | --- |
| **Hysterectomy** | All 68, all 69, (excl. 695-699), all 653, all 654, all 655, all 656, all 664, all 665, all 667 |
| **Prostatectomy** | 603, 604, 605, 606, |
| **Nephrectomy** | All 553, all 554, all 555 |
| **Pulmonary lobectomy** | All 32 |
| **Cardiac surgery** | All 36, 35 and 37 |
| **Colectomy** | All 48, all 457, 452 all 49, all 458 |
| **Cystectomy** | 576. all 577 |
| **TransOral Robotic Surgery (TORS)** | 8131, 8101, 066, all 27, all 28, all 29, all 30, 316 |
| **Hepatectomy** | All 50 (excl., 505, 506, 509) |
| **Thyroidectomy** | 060, 061, 062, 063, 064, 065, 066, 064 |

In addition to this filter, we also used the secondary procedure fields (from PR2 to PR15) to extract hospital discharges that relied upon one or more secondary robotic procedures identified through the codes reported in S10 Table.

**S10 Table.** ICD-9-CM codes for the selection of robotic secondary procedures in NIS**®** 2012.

|  | **ICD-9-CM Codes for secondary procedures** |
| --- | --- |
| **Robotic-assisted procedures** | 1741, 1742, 1743, 1744, 1745, 1749 |

S11 Table shows the rank of the ten surgical robotic procedures performed in 2012 as recorded in the NIS**®** dataset.

**S11 Table.** Number of surgical procedures performed in 2012 according to the NIS**®** dataset.

| **Surgical procedure** | **Number of robotic procedures performed (NIS®)** |
| --- | --- |
| **Hysterectomy** | 9,949 |
| **Prostatectomy** | 8,027 |
| **Nephrectomy** | 2,673 |
| **Pulmonary lobectomy** | 844 |
| **Cardiac surgery** | 634 |
| **Colectomy** | 615 |
| **Cystectomy** | 419 |
| **TransOral Robotic Surgery (TORS)** | 145 |
| **Hepatectomy** | 55 |
| **Thyroidectomy** | 25 |

# **S3 Results**

# **S3.1 Robustness of the rankings by size, structural depth, and structural width**

In order to assess the robustness of our analysis based on of the ranking of surgical specialties according to the median of the distributions of cascade size, structural depth and structural width, we quantified the Kendall rank correlation coefficients to measure the similarity between the median-based rankings and alternative rankings obtained using different descriptive statistics of the same distributions, including the 75^th^ percentile, the Coefficient of Variation (CV), the quartile coefficient of dispersion, the Median Absolute Deviation (MAD) and its normalized version, the Mean absolute Difference (MD), and the Relative Mean absolute Difference (RMD). S12 Table reports results based on those tests, and indicates that, across all alternative descriptive statistics, observations have a statistically significantly similar rank.

**S12 Table.** Kendall’s tau correlation coefficients. Tests of independence between rankings of the ten surgical specialties obtained using the median of their distributions of cascade size, structural depth and width, on the one hand, and the rankings based on other selected descriptive statistics. *: rank correlation coefficient is statistically significant at the 0·05 level; **: rank correlation coefficient is statistically significant at the 0·01 level (2-tailed tests). CV = Coefficient of Variation; MAD = Median Absolute Deviation (MAD); MD = Mean absolute Difference (MD); RMD = Relative Mean absolute Difference.

| **Descriptive statistic** | **Kendall’s tau correlation coefficient (size)** | **Kendall’s tau correlation coefficient (structural depth)** | **Kendall’s tau correlation coefficient (structural width)** |
| --- | --- | --- | --- |
| **75th percentile** | 0.719** (p=0.005) | 0.778** (p=0.0009) | 0.750** (p=0.004) |
| **CV** | -0.045 (p=0.928) | -0.689** (p=0.005) | 0.295 (p=0.279) |
| **Quartile coefficient of dispersion** | 0.629* (p=0.015) | 0.644** (p=0.005) | -0.367 (p=0.241) |
| **MAD** | 1** (p=0.0001) | 0.822** (p=0.004) | 0.614* (p=0.019) |
| **Normalized MAD** | 0.744** (p=0.005) | 0.778** (p=0.0009) | -0.735** (p=0.005) |
| **MD** | 0.629* (p=0.015) | 0.733** (p=0.002) | 0.796** (p=0.002) |
| **RMD** | 0.089 (p=0.787) | 0.689** (p=0.005) | -0.341** (p=0.207) |

# **S3.2 Tests of independence between distributions of level of evidence**

Fig 6 (panel f) in the main text reports our findings on the ranking by innovation index. We noted that the ranking comes as no surprise. For instance, robotic cardiac surgery is increasingly employed as it offers a minimally invasive approach for coronary revascularization and other procedures such as valve repair and closure of atrial septal defect through a closed chest [8, 9]. In contrast, TORS, though a potentially promising treatment modality for oropharyngeal cancer and obstructive sleep apnoea, for now lacks the high-level evidence needed to support its use against traditional treatments (chemoradiotherapy and continuous positive airways pressure, respectively). In addition, the majority of the robotic instruments (not originally designed for transoral surgery) limit the applicability of TORS to a select minority of patients [10, 11].

For each surgical specialty, S13 Table reports the number of articles associated with level of evidence, the number of seed articles generating cascades, and the fraction of seed articles with an associated level of evidence. The table suggests that not all cited articles across all specialties can be placed within the surgical innovation funnel.

**S13 Table.** Count of cascades associated with level of evidence across surgical specialties.

| **Specialty** | **Count of articles with**  **level of evidence** | **Count of cascades (i.e., seed articles)** | **Coverage (%)** |
| --- | --- | --- | --- |
| **Prostatectomy** | 623 | 835 | 72% |
| **Cardiac surgery** | 310 | 374 | 83% |
| **Nephrectomy** | 239 | 279 | 86% |
| **Hysterectomy** | 180 | 194 | 93% |
| **Cystectomy** | 116 | 149 | 78% |
| **Thyroidectomy** | 77 | 87 | 89% |
| **Colectomy** | 61 | 79 | 77% |
| **Pulmonary lobectomy** | 57 | 70 | 81% |
| **TransOral Robotic Surgery (TORS)** | 44 | 48 | 92% |
| **Hepatectomy** | 37 | 44 | 84% |

S1 Fig shows the distributions of ordinal categories associated with the implementation stages according to levels of evidence for each surgical specialty. For each of the 45 possible pairs of surgical specialties, we performed a two-way Chi-squared test of independence between the corresponding distributions of levels of evidence (S1,a Fig). The distributions of prostatectomy and cardiac surgery show a statistically significant difference with respect to all other procedures except one (thyroidectomy and hepatectomy, respectively). For robustness check, we also performed a Mann-Whitey U test of independence for each of the 45 pairs of specialties (S1,b Fig). The null hypothesis of lack of independence is rejected when p-value<0.05. Both tests produce very similar results, with only few exceptions.


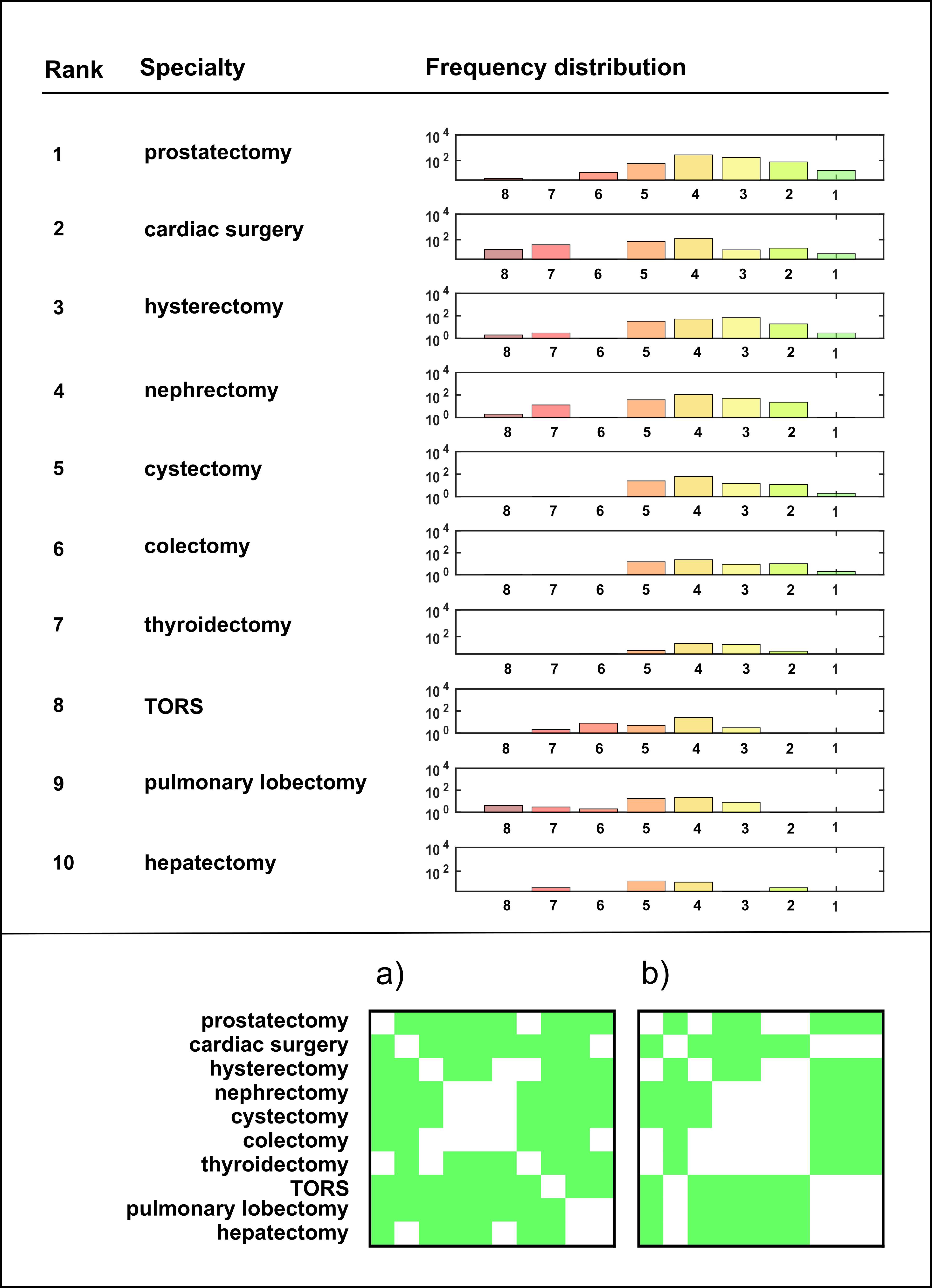


**S1 Figure.** **Frequency distributions of implementation stages and independence tests.** Top panel: distributions of ordinal categories associated with implementation stages according to level of evidence for each surgical specialty. Bottom panel: a) two-way Chi-squared tests of independence and b) Mann-Whitney U tests of independence between distributions of implementation-based categories. Green squares correspond to values of the test statistic that reject the null hypothesis of non-independence with p-value<0.05.

# **S3.3 Spearman’s and Kendall’s tau tests for correlation between rankings**

We calculated Spearman’s rank correlation coefficients (S14 Table) and Kendall’s tau coefficients (S15 Table) to compare rankings of surgical specialties by number of citations to seed articles, fraction of citations within one year, cascade size, structural depth, structural width, innovation index, and count of robotic procedures in NIS**®**. The results suggest that the rankings according to cascade size and structural depth are statistically significantly similar to the ranking by innovation index. In contrast, the ranking of surgical specialties by number of citations does not correlate significantly with either of the other rankings. Notice that results from Kendall’s tau tests are consistent with the results reported in the main text.

**S14 Table.** Spearman’s rank correlation coefficients with exact p-values testing the independence between several pairs of rankings of surgical specialties.

|  | **Innovation index** | **Number of procedures performed (NIS®)** |
| --- | --- | --- |
| **Citations** | 0.236  (p*=*0.514) |  |
| **Fraction of citations** | -0.188  (p*=*0.607) |  |
| **Cascade size** | 0.758*  (p*=*0.016) |  |
| **Structural depth** | 0.782*  (p*=*0.012) |  |
| **Structural width** | 0.624  (p*=*0.060) |  |
| **Innovation index** |  | 0.673*  (p*=*0.039) |

Note**:** *: p-value<0.05; **: p-value<0.01; ***: p-value<0.001.

**S15 Table.** Kendall’s tau correlation coefficients with exact p-values testing the independence between several pairs of rankings of surgical specialties.

|  | **Innovation index** | **Number of procedures performed (NIS®)** |
| --- | --- | --- |
| **Citations** | 0.2  (p*=*0.484) |  |
| **Fraction of citations** | -0.156  (p*=*0.601) |  |
| **Cascade size** | 0.556*  (p*=*0.027) |  |
| **Structural depth** | 0.511*  (p*=*0.047) |  |
| **Structural width** | 0.378  (p*=*0.156) |  |
| **Innovation index** |  | 0.511*  (p*=*0.047) |

Note: *: p*<*0.05; **: p*<*0.01; ***: p*<*0.001.

The ranking of specialties by our measure of innovation index was found to closely match the one based on the NIS**®** dataset. This comes as no surprise. For example, robotic prostatectomy has long been the commonest approach for prostate cancer in the US but increasingly also in the UK and Europe supported by level 1 evidence of superiority over traditional approaches [12]. In contrast, robotic thyroidectomy is a procedure originally developed in the Far East primarily to address cultural concerns relating to the presence of neck scar with a remarkably poor uptake in the Western world [13].

**S3.4 Concordance correlation coefficient and adjustment for multiple comparisons**

We conducted further statistical analysis, and evaluated multiple comparisons with Kendall’s *W* concordance coefficient [14]. The Kendall *W* concordance coefficient computes the association between *N* sets of rankings of *k* items, and bears a close relationship with the Friedman’s test. In our case, the concordance coefficient measures the extent to which there is agreement between pairs of rankings of the ten surgical procedures based on our six different criteria (citations, depth, etc.). The Kendall concordance ranges from 0 to +1. Values close to zero represent lack of agreement (or perfect disagreement) between the rankings of items, while values close to one represent perfect agreement in the rankings.

S16 Table reports values of the Kendall’s W concordance coefficient with exact p*-*values. The null hypothesis is that there is no concordance (i.e., that there is independence) between pairs of rank distributions of procedures, each based on one of our six measures. As suggested by the table, the null hypothesis is once again rejected when the ranking based on innovation value is compared to rankings based on cascade size, structural depth, and structural width. In all other cases, the null hypothesis cannot be rejected, and the test thus suggest that surgical procedures are ranked differently when the criteria are innovation value, citations count, and fraction of citations within one year.

**S16 Table**. Kendall’s concordance coefficients and exact p*-*values for tests of independence between pairs of rankings of ten surgical procedures.

|  | **Fraction of citation by 1 year** | **Size** | **Structural depth** | **Structural width** | **Innovation**  **index** |
| --- | --- | --- | --- | --- | --- |
| **Citations** | 0.636 (p=0.224) | 0.636 (p=0.224) | 0.515 (p=0.473) | 0.515  (p =0.473) | 0.618 (p=0.257) |
| **Fraction of citation by 1 year** |  | 0.285 (p=0.898) | 0.279 (p=0.904) | 0.267 (p=0.904) | 0.406 (p=0.708) |
| **Size** |  |  | 0.958*** (p=0.000) | 0.964*** (p=0.000) | 0.879**  (p =0.007) |
| **Structural depth** |  |  |  | 0.982*** (p=0.000) | 0.891** (p=0.005) |
| **Structural width** |  |  |  |  | 0.812* (p=0.030) |

Note: *: p*<*0.05; **: p*<*0.01; ***: p*<*0.001.

Finally, we calculated exact p*-*values by adjusting for multiple comparisons based on false discovery rate (FDR) correction. Once again, the null hypothesis is that the rankings are independent. The hypothesis is therefore rejected when the rankings are in agreement [15, 16]. Results are reported in S17 Table.

**S17 Table**. Kendall’s concordance coefficients and exact p*-*values adjusted with the FDR method for multiple (i.e., 5) comparisons for tests of independence between pairs of rankings of ten surgical procedures.

|  | **Innovation index** |
| --- | --- |
| **Citations** | 0.618  (p=0.321) |
| **Fraction of citation by 1 year** | 0.406  (p=0.708) |
| **Size** | 0.879**  (p =0.017) |
| **Structural depth** | 0.891**  (*p*=0.017) |
| **Structural width** | 0.812*  (p*=*0.050) |

Note: *: p*<*0.05; **: p*<*0.01; ***: p*<*0.001.

**References**

1. Jacomy M, Venturini T, Heymann S, Bastian M. ForceAtlas2, a continuous graph layout algorithm for handy network visualization designed for the Gephi software. PLoS One. 2014; 9(6):e98679.

2. Kempe D, Kleinberg J, Tardos, E. Maximizing the spread of influence through a social network. Proceedings of the ninth ACM SIGKDD international conference on Knowledge discovery and data mining. 2003; 137-46.

3. Wiener H. Structural determination of paraffin boiling points. J Am Chem Soc. 1947; 69(1):17-20.

4. Goel S, Anderson A, Hofman J, Watts DJ. The Structural Virality of Online Diffusion. Management Science. 2016; 61(1):180-96.

5. Hutchison K, Rogers W, Eyers A, Lotz M. Getting Clearer About Surgical Innovation: A New Definition and a New Tool to Support Responsible Practice. Ann Surg. 2015; 262(6):949-54.

6. Dunphy SM, Herbig PR, Howes ME. The Innovation Funnel Technological Forecasting and Social Change. 1996; 53:279-92.

7. Hansen MT, Birkinshaw J. The Innovation Value Chain. Harvard Business Review. 2007; 85(6):121-30.

8. Kim JE, Jung SH, Kim GS, Kim JB, Choo SJ, Chung CH, et al. Surgical Outcomes of Congenital Atrial Septal Defect Using da Vinci^TM^ Surgical Robot System. Korean J Thorac Cardiovasc Surg. 2013; 46(2):93-7.

9. Whellan DJ, McCarey MM, Taylor BS, Rosengart TK, Wallace AS, Shroyer AL, et al. Trends in Robotic-Assisted Coronary Artery Bypass Grafts: A Study of The Society of Thoracic Surgeons Adult Cardiac Surgery Database, 2006 to 2012. Ann Thorac Surg. 2016; 102(1):140-6.

10. Arora A, Chaidas K, Garas G, Amlani A, Darzi A, Kotecha B, et al. Outcome of TORS to tongue base and epiglottis in patients with OSA intolerant of conventional treatment. Sleep Breath. 2016; 20(2):739-47.

11. Arora A, Kotecha J, Acharya A, Garas G, Darzi A, Davies DC, et al. Determination of biometric measures to evaluate patient suitability for transoral robotic surgery. Head Neck. 2015; 37(9):1254-60.

12. Robertson C, Close A, Fraser C, Gurung T, Jia X, Sharma P, et al. Relative effectiveness of robot-assisted and standard laparoscopic prostatectomy as alternatives to open radical prostatectomy for treatment of localised prostate cancer: a systematic review and mixed treatment comparison meta-analysis. BJU Int. 2013; 112(6):798-812.

13. Arora A, Garas G, Sharma S, Muthuswamy K, Budge J, Palazzo F, et al. Comparing transaxillary robotic thyroidectomy with conventional surgery in a UK population: A case control study. Int J Surg. 2016; 27:110-7.

14. Kendall MG, Babington Smith B. The Problem of m Rankings. The Annals of Mathematical Statistics. 1939; 10:275-87.

15. Legendre P. Coefficient of concordance. In: Salkind NJ, editor. Encyclopedia of Research Design. 1. Los Angeles, CA: SAGE Publications, Inc.; 2010. p. 164-9.

16. Pike N. Using false discovery rates for multiple comparisons in ecology and evolution. Methods in Ecology & Evolution. 2010; 2(3):278-82.
